# Insights on conducting digital patient and public involvement in dementia research during the COVID-19 pandemic: supporting the development of an “E-nabling digital co-production” framework

**DOI:** 10.1186/s40900-022-00371-9

**Published:** 2022-07-26

**Authors:** Mauricio Molinari-Ulate, Rebecca Woodcock, Isabelle Smith, Henriëtte G. van der Roest, Manuel A. Franco-Martín, Michael P. Craven

**Affiliations:** 1 Psycho-Sciences Research Group, Institute of Biomedical Research of Salamanca, University of Salamanca, Salamanca, Spain; 2 Department of Research and Development, Iberian Institute of Research in Psycho-Sciences, INTRAS Foundation, Zamora, Spain; 3 NIHR MindTech MedTech Co-Operative, Institute of Mental Health, University of Nottingham, Nottingham, UK; 4 NIHR Nottingham Biomedical Research Centre, University of Nottingham, Nottingham, UK; 5 Department on Aging, Netherlands Institute of Mental Health and Addiction (Trimbos Institute), Utrecht, The Netherlands; 6 Psychiatric and Mental Health Department, Zamora Healthcare Complex, Zamora, Spain; 7 Human Factors Research Group, Faculty of Engineering, University of Nottingham, Nottingham, UK

**Keywords:** Dementia, COVID-19, Patient and public involvement, PPI, Dementia research, Co-production

## Abstract

**Background:**

The rapid transition to digital working, accelerated due to the response to the COVID-19 pandemic, has impacted the involvement of patients and public in research. This paper presents experiences of engaging in digital Patient and Public Involvement (e-PPI) in dementia research since the lockdowns, offering recommendations regarding future digital and hybrid working. Furthermore, it introduces a co-produced framework for researchers, PPI coordinators and public contributors to identify and discuss challenges and opportunities provided by e-PPI.

**Methods:**

Two online workshops and one individual interview were performed with a group of researchers and PPI coordinators with experience in PPI in dementia research, and with an existing dementia PPI group having some experience of working online during the pandemic. The project was constructed as a PPI activity, with the MindTech Involvement Team (PPI group) involved in the entire process, and a collaborative data analysis process was adopted.

**Results:**

After refinement of the coding structure, the MindTech Involvement Team and Project Leaders identified four main themes, resulting in the ‘E-nabling Digital Co-production' Framework. During this framework development, different positions were expressed, associated with the transition to digital working. Two main themes were shared by the participating groups regarding e-PPI: wider potential reach without geographical constraints, and the perception of more business-like sessions with reduced opportunities for social interactions and communication. Specifically for dementia research, whilst e-PPI may allow public contributors to attend more meetings, potentially mutually supportive environments provided by face-to-face meetings could be diminished, with carers experiencing a possible reduction in informal respite opportunities.

**Conclusions:**

Through involving public contributors, researchers, and PPI coordinators with a focus on digital PPI in dementia research, we were able to further refine and co-produce the ‘E-nabling Digital Co-production' Framework. Demonstrating potential for analysis of benefits and limitations within e-PPI, it was possible to identify both general insights and those specific to dementia research. However, the most significant contribution of the framework is the potential to support local journeys of co-production in ongoing digital and hybrid public involvement activities.

**Supplementary Information:**

The online version contains supplementary material available at 10.1186/s40900-022-00371-9.

## Abstract (summary)

The COVID-19 pandemic has impacted the engagement of patients and the public in research. Lockdowns, social distancing, and reduced physical contact have affected the involvement of public contributors in research studies. In particular, the pandemic triggered a rapid transition to digital working, increasing the use of Information and Communication Technologies such as video conferencing on computers and mobile devices. With little time to reflect on the consequences of digital working in PPI and with a continuing legacy of hybrid or blended approaches to involvement, this project highlights the challenges and potential for e-PPI approaches (electronic/digital PPI) within the context of dementia research. In addition to examining the transition to digital working in this area, we present a co-produced framework for researchers, PPI coordinators and public contributors.

**Supplementary Information:**

The online version contains supplementary material available at 10.1186/s40900-022-00371-9.

## Background

Patient and Public Involvement (PPI) has gained more attention in recent years across all areas of health research [1], including dementia [2, 3]. Considered as a cornerstone for governmental and ethical policies in health research along with the development of PPI best practice guidelines [2, 4, 5] it has been defined as a research project or public policy development carried out with or by patients or members of the public that is beyond their engagement as subjects [5–7].

With practical benefits in enhancing the quality of the research [3–5] and as part of an accepted discourse [8], PPI occupies at minimum a stipulated requirement, rather than an option, including funding applications for health research [5]. Whilst democratic rationales [9, 10] may receive less attention than technocratic or transactional motivations, patient involvement has the potential to either address or exacerbate existing inequalities within health outcomes [11]. Indeed, these existing inequalities risk being further compounded through the COVID-19 pandemic [12].

Since the beginning of the pandemic, declared by the World Health Organization in March 2020, the involvement of patients and the public in research has been challenged because of social distancing, lockdowns, and other reduced physical contact [13]. Therefore, quick responses and adjustments have been needed, which have been accompanied by increased use of Information and Communication Technologies (ICT) [14, 15]. With the definition, breadth and theoretical underpinning of PPI already conceptually challenged and contested [9] alongside a range of practices and values underpinning its delivery [16], the move to digital represents a further domain in which complicated dynamics exist.

Previous literature on conducting digital PPI (referred to henceforth as e-PPI) is scarce [7, 17], however, it highlights several challenges that differ from those found in face-to-face meetings such as: (a) less spontaneous interactions between the individuals (e.g., more direction from the meeting chairs, breaks taken individually), (b) lack of non-verbal cues (e.g., difficult to observe non-verbal communication such as gestures), (c) difficulties in turn-taking (e.g., less spontaneous change of speaker), (d) changes in the meeting chair role (e.g., a more active and directive role), (e) linguistic barriers (e.g., less participation in discussions), or (f) limited view of each participant’s face [17]. Also, a scoping review looking at the role of ICT to involve patients and the public identified limitations of internet use as being impersonal, expensive, or stressful, and it was considered that weblogs cannot be a replacement for in-person meetings [7]. Wider consideration of approaches to understand Working From Home (WFH), whilst not focused on PPI, may also serve to highlight relevant factors [18].

As COVID restrictions have gradually eased in some countries this has led to more hybrid approaches such as blended meetings (online contributions and face-to-face attendance). With such a legacy, the importance of getting e-PPI right will remain a topical and evolving issue.

### Co-producing an immediate local response

MindTech (https://www.mindtech.org.uk) is a national centre established in 2013 and funded by the National Institute for Health and Care Research (NIHR) focussing on the development, adoption, and evaluation of new technologies for mental healthcare and dementia. The MindTech Involvement Team, a group of people and carers bringing their own lived experiences of mental health conditions, as well as expertise in the processes of patient and public involvement, occupies a strategic and advisory role in the organisation, aiming to involve patients and public in all aspects of research.

Experiencing an immediate and instrumental shift to e-PPI in March 2020, the MindTech Involvement Team continued to meet regularly through virtual meetings despite not previously operating online involvement methods. Although public contributors, staff (employed as PPI coordinators) and researchers acknowledged inherent challenges and opportunities that this brought to ensuring continued meaningful involvement, there was a recognition that these would differ at individual, group, and research level. As a localised response, the MindTech Involvement Team and PPI staff co-produced an overview of the primary areas that were impacted by the shift to e-PPI. Presented at the MindTech Symposium in December 2020, these considerations were accompanied by a set of local actions taken to both mitigate challenges and harness the potential from these new ways of working (Additional file 1: Overview of Digital Considerations).

From developing an initial generic set of considerations regarding e-PPI, the work continued with a project conceived in November 2020 as part of the first author’s (MMU) training programme placement for the European DISTINCT network (https://www.dementiadistinct.com). Working remotely in collaboration with the local academic supervisor from MindTech and the Centre for Dementia at the Institute of Mental Health (MPC), MindTech Involvement Team staff PPI co-ordinator (RW) and with a designated public contributor from within the team as project co-lead (IS), this afforded the opportunity for further involvement and application of these considerations within the context of dementia-specific PPI.

Whilst PPI groups nationally grappled with many similar elements, systematic consideration and understanding of the relative successes of different PPI groups within the transition to e-PPI remained elusive. It was proposed that although there remained a set of shared considerations, success may be predicated on both the area of health research and significantly on the type of group experiencing the transition to online involvement.

People Living with Dementia (PLwD) and carers represent one such group. Although far from homogenous, PPI groups working with PLwD may share a set of challenges and opportunities with respect to this digital transition and which are additional to considerations already well articulated [2]. With telephone or e-mail already identified as valuable tools for PPI representatives’ engagement in dementia research [6], further insights may be made through exploring the experiences of using ICT, with a view to identifying better approaches for public involvement and making the most of *experts by experience.*

We therefore decided to explore the experiences of e-PPI within a dementia-specific context during the COVID-19 pandemic and intended to use the findings to refine the existing ‘Overview of Digital Considerations’ (Additional file 1) originally developed by the MindTech Involvement Team, resulting in an ‘E-nabling Digital Co-production’ Framework. The framework is introduced as a tool for researchers, PPI coordinators and vitally public contributors themselves to identify and discuss challenges and opportunities provided by e-PPI and future blended and hybrid approaches.

## Methods

### Developing a Co-produced Dementia e-PPI project

Continuing the focus on co-production, the project leads ran three types of session: (a) a project design and development session, (b) project delivery sessions (workshops), and (c) meetings to analyse and synthesis the outcomes. One representative member of the Involvement Team was a co-lead of the project and was involved as a facilitator of the online workshops (IS).

### Online workshops

The project co-leads selected by preference a workshop approach as opposed to alternatives such as semi-structured interviews with individual PPI members, considering it the most pragmatic strategy to working online with PPI groups. Workshop formats allow an exchange of ideas within a scaffolded structure, inclusion of potential challenges or allow for a range of positions expressed within a supported environment, thereby enabling various positions within a group forum to be identified. By undertaking workshops online, it is also possible to share comments through the chat function, where a parallel discussion can be facilitated, allowing people to share their thoughts without having to speak to the rest of the group.

Before each of the sessions, participants were provided with a project information sheet and a semi-structured guide of possible topics and questions to cover at the discussions (Additional files 2 and 3, respectively). A one-minute pre-recorded pitch was shown at the beginning of each of the sessions by way of introducing the project, inviting individuals to participate, and as an “ice-breaking” strategy to initiate the activity. The time for the sessions varied from 25 min to approximately an hour.

Two online workshops and one individual interview (the latter for one academic researcher who could not attend the workshops) were conducted. Two roles were provided by the project co-leads: (a) facilitation of the workshops and discussion (MMU and IS); and (b) administrative and inclusion role, with a person in charge of taking field notes and checking the chat box (RW and IS). Field notes were chosen to gather the information as they have been previously implemented in similar public engagement projects [19, 20] and because verbatim transcripts would not be available, since the sessions were not recorded to maintain the policies of the PPI groups involved and as this project was organised as a PPI rather than a research activity (see ethical approval statement). The online platform used was Microsoft Teams (MS Teams) as this was the tool facilitated by the institutions involved.

### Groups participating in the workshops

To develop a broader perspective on the challenges and experiences related to the transition to e-PPI in dementia research, we contacted researchers and PPI coordinators (either staff or public contributors that has a role in facilitating PPI) for one of the workshops. Four researchers and two PPI coordinators accepted the invitation and were invited to a group session (Workshop 1). An individual interview was held with one of the researchers as accepted the invitation but could not attend the workshop.

A second workshop was performed with an existing PPI group, the ‘Dementia, Frail Older People and Palliative Care Patient and Public Involvement Advisory Group’ (the Advisory Group from now on) from the University of Nottingham (Workshop 2). The Advisory Group is made up of members who have experience of caring for PLwD, are carers themselves, who provide advice and guidance at all stages of research projects. The group was meeting regularly once a month and a request to participate in one of their sessions was sent by MMU. The workshop strategy was brought to one of the Advisory Group’s existing virtual meetings and a total of 11 members were part of this session. Using an existing PPI group provided a safe and structured settings for working with PLwD carers, recognising the need for increased attention to ethical and welfare issues as described in the literature [2].

These two groups participated only in their respective workshop sessions and were not involved in any other stage of the project.

### Qualitative analysis method

**Fig. 1:**
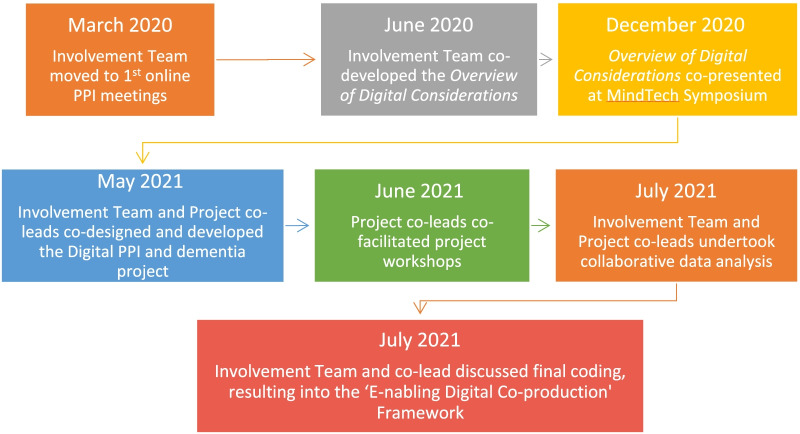
The co-production journey of the “E-nabling Digital Co-production” Framework

A thematic analysis was the chosen approach for the analysis of the results. In keeping with a methodological approach based on co-production, a collaborative data analysis (CDA) was performed with members of the MindTech Involvement Team [21]. The co-leads (IS and RW) and other members of the Involvement Team held an online meeting session to start coding the information, identifying the potential to utilise the Overview of Digital Considerations document (Additional file 1) to support this endeavour. After initially reviewing the initial coding co-production continued with our public contributor project co-lead (IS) and the other project leads (MMU and RW) working together, leading to consolidation into four key themes. This included addition of the concept of ‘involvementability’ as identified within the researchers and PPI coordinators Workshop. This resulted in the ‘E-nabling Digital Co-production' Framework (see Fig. 1). MMU, RW and IS continued with the final coding before ambiguities and final coding was brought back to the Involvement Team for discussion and final inputs.

**Fig. 2:**
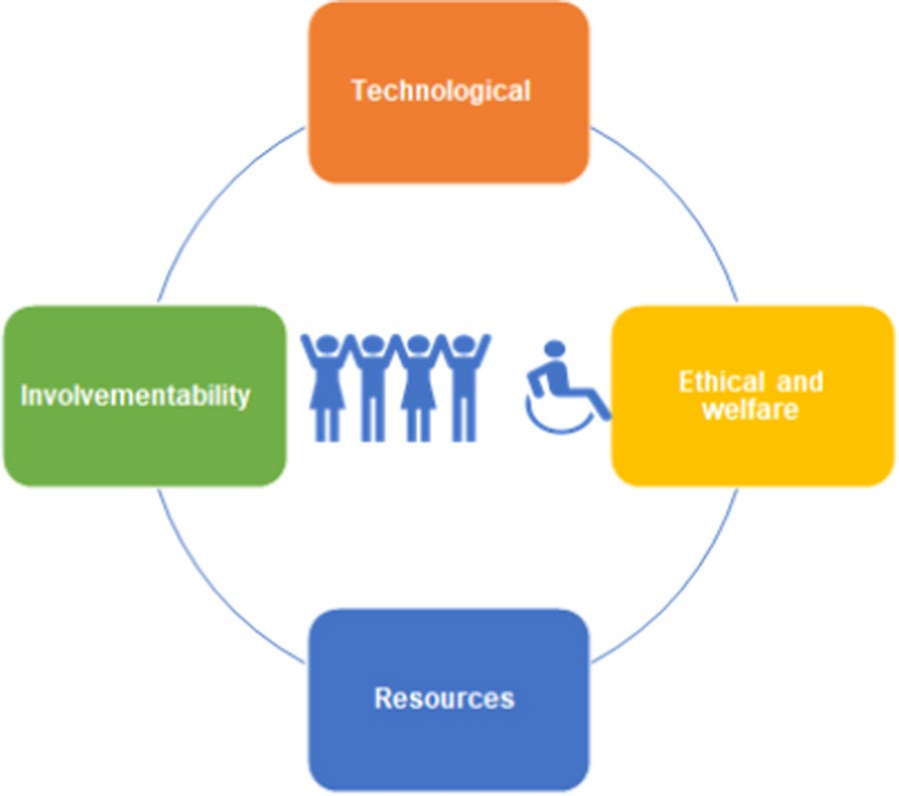
“E-nabling Digital Co-production” Framework

An overview of the ‘E-nabling Digital Co-production' Framework can be seen in Fig. 2, with descriptions of each of the four areas of the framework available in Table 1.

**Table 1:** Description of the four key areas of the “E-nabling Digital Co-production” Framework

The ‘E-nabling Digital Co-production’ Framework	
Key area	Description
Technological	Technological considerations include assessment of the constraints, preferences, and opportunities that technology can provide **Preferences:** ● How are preferences and any support needs identified by public contributors communicated with researchers? **Power:** ● What is the potential for shared decision-making regarding the use of technology, including functional and operational components? ● To what extent are technological considerations revisited regularly with public contributors recognising the fast pace of developments in online collaborative platforms? ● What potential exists for supporting researchers, PPI staff and public contributors to develop confidence in using online methods?
Resources	Resources were considered at a personal or a more mechanistic level **Personal:** ● Consideration of increased emotional toll with online involvement, with recognition of increasing fatigue and additional personal resilience often required for negotiating challenging work within a virtual context **Professional resources:** ● Both payment for lived experience input and increased demands on those delivering PPI online **Preparation:** ● Are additional requirements planned from the outset? These could include additional facilitation roles, onboarding sessions, costs of coproduction platforms, phone credit/printing etc./software, budgeting for an increased frequency but shorter meetings ● Wider resources may include additional training for researchers, PPI staff and public contributors to support the use of new technology
Involvementability	‘Involvementability’ is offered as an example of a non-functional requirement, a concept that aims to describe requirements that are related to the success of a design task or process but are not integral to its content [22] **Process:** ● How does the nature of involvement method or process itself impact on the extent that meaningful involvement can be achieved? ● How do codesign methods differ in a digital space? **Product:** ● How does the area of health research itself impact on the extent that involvement can easily translate to a digital space, such as exploring digital health interventions may be facilitated or made more complex through online involvement? **Population:** ● How easily will ‘involvement’ translate online for different populations?
Ethical and Welfare	How does digital PPI interact with a range of areas including: ● Welfare of public contributors ● ﻿Digital exclusion ● Impact of digital engagement on social communication ﻿● Power ● Safeguarding ● Privacy, confidentiality, and data security

## Results

The workshops were held in June 2021 and 14 pages of notes resulted from a total meeting time of 2 h.

To explore the move to e-PPI for dementia research during the pandemic, the insights from the workshops were mapped against the four areas of the ‘E-nabling Digital Co-production’ Framework, as it was refined following the co-production journey described previously. This approach also allowed for the opportunity to identify and highlight specific insights for dementia research.

The following section demonstrates how insights were then categorised according to the co-produced framework themes. Both positive and negative aspects of e-PPI were expressed by the participants and recorded accordingly. Dementia specific remarks were highlighted separately for each theme.

### Technological

All remarks about technology aspects came from the researchers and coordinators group. Three main technical issues were highlighted: online platform alternatives, technical support, and accessibility. Concerning access to different platforms, researchers and coordinators commented on restrictions due to institutional rules or policies which determine the platform options, such as requiring use of MS Teams rather than a broader choice. Other platforms were identified as having potential to solve various barriers or constraints to participation, while some individuals were more familiar with or preferred different platforms.

Other reflections emphasised the need for technical or administrative support having responsibility during virtual meetings to help resolve technical issues, and the concern about difficulties identified in giving full access to other participants.

### Resources

A diversity of topics arose about resources. On the positive side, there was time saved on not travelling to meetings, with implications particularly for public contributors who are most often asked to attend meetings in research settings away from their homes. For researchers and PPI coordinators, facilitating arrangements in terms of venues, catering, or other coordination, such as transportation for PPI representatives was identified as resource intensive. However, it was understood that other resource requirements may be needed instead, such as the time for additional support staff to facilitate online delivery, or the practice of providing additional reimbursement to recompense costs incurred through online working. Another positive was the reflection that virtual meetings were more “straight to the point” although this may itself contribute to some of the perceived lack of informal communication and connections that face-to-face PPI may create. Also mentioned was that online meetings allowed members to attend more meetings. Finally, both researchers and coordinators, and the members of the Advisory Group, considered that e-PPI had a wider potential reach, with virtual meetings enabling connections with researchers or participants that are geographically dispersed. On the negative side, attendees underlined that planning e-PPI is more time consuming, and others mentioned that controlling time and contributions could be harder.

Particularly for carers of PLwD, it was considered that virtual meetings helped to overcome some of the limitations related to their role as carers, such as concerns around time away, finding an alternative carer, or other time constraints, as they could attend the PPI sessions from home.

### Involvementability

Three main barriers for e-PPI involvement stood out regarding this area: virtual meeting limitations, communication, and social interaction.

*Virtual meeting limitations*: researchers and coordinators emphasised the limitations of the type of activities that could be done; some of the insights referred to the difficulties in involving individuals when different devices are required. One example was the need for multiple devices as in the case of using a laptop for ICT while the research was to evaluate another device or software, such as an app on a phone or tablet. Another issue arising from the use of video communications, mentioned by researchers, is the limited view of physical prompts or other non-verbal communication.

*Communication*: both groups considered that communication is less effective during virtual meetings. Reflections that reinforced this idea related to feedback mechanisms and interchanges between attendees and researchers that were missing or diminished in an online exchange. It was proposed that this was affected by the reduced non-verbal communication, resulting in less fluid discussion (e.g., more formal turn-taking) and not being able to see all participants on the screen at the same time. However, a positive reflection from researchers and coordinators was that virtual meetings served to encourage reflection about communication methods, particularly the role of raising hands and waiting for an opportunity to participate.

One area of potential ambiguity was whether e-PPI served to increase the inclusion of those less confident in participating. Whilst participation could be more easily regulated with facilitation leading to greater inclusion, it was also highlighted that online interactions may create or reinforce additional barriers to engagement.

Specifically for dementia research, participants underlined the need to consider cognitive abilities as the level of attention or concentration needed for virtual meetings could affect the discussion and engagement, for example, someone may forget their contribution by the time they have an opportunity to talk.

*Social interaction*: overall participants had experienced less social interaction through virtual meetings. They mentioned a decrease of informal social interaction, such as breaks during the sessions that allow for spontaneous conversations and interchange between attendees, and lack of opportunities to share and meet with others, leading to a more “business-like” meetings.

In terms of positive contributions, researchers and coordinators considered that e-PPI could be less threatening concerning the physical social interaction and that, normally, the individual will be participating in a safe environment (e.g., their home). Additionally, they mentioned that people wishing to isolate for any reason (e.g., COVID) can be included, and that close and strong relationships could be developed.

Regarding dementia specific remarks, it was considered that meeting online may diminish the opportunity of a respite and supportive space for members providing care, which seems to be accomplished in face-to-face meetings.

### Ethical and welfare

The following three main topics were identified in this area:

*Diversity and inclusion*: as a negative perception, researchers and coordinators considered that e-PPI could be a barrier for inclusion as the group of individuals attending virtual meetings may stay the same without new members coming along. Also, they considered that the group does not represent all sectors of the community.

On the contrary, virtual environments could offer the opportunity to include those that have not been considered for several circumstances. However, it was noted that to achieve this, recruitment methods would need to be improved with further recognition that this is compounded, with increased difficulties in recruiting PLwD in an online context.

*Digital inclusion*: several barriers were identified by members from the Advisory Group, including that with e-PPI, some individuals could feel that they are not part of the research team as the sense of group is missing, and that difficulties with the technology or the dislike for virtual meetings was a factor in losing participants. A similar barrier was considered by researchers and coordinators as they mentioned that e-PPI could be excluding individuals that lack the skills and confidence needed to use the technology, which might be exacerbated in vulnerable populations, and that even those familiar with the platforms struggled when technical issues occurred.

Furthermore, equity arose as a concern in terms of the technology use and support, particularly because some individuals could have better access while others do not (e.g., good bandwidth) and those in need of support or living alone might not be able to join (e.g., PLwD).

Regarding dementia specific remarks, the participation and presence of the caregiver is harder to distinguish in virtual meetings, with more sophisticated facilitation skills needed to support meaningful participation of PLwD and carers.

*Ethical issues*: some of the barriers mentioned by researchers and coordinators related to the need for clarity regarding reimbursement of public contributors participating remotely, gaining informed consent to record virtual meetings, and providing emotional support when people get distressed or frustrated.

Specifically for dementia, the severity of cognitive impairment arose as a consideration. As cognitive function determined the level of support needed at the virtual meetings, this was not always straightforward to assess or address. Furthermore, it was identified that there was an increased difficulty in determining levels of caregiver support and input, with potential to diminish participation from the individual living with dementia.

### Tips to improve e-PPI

**Fig. 3:**
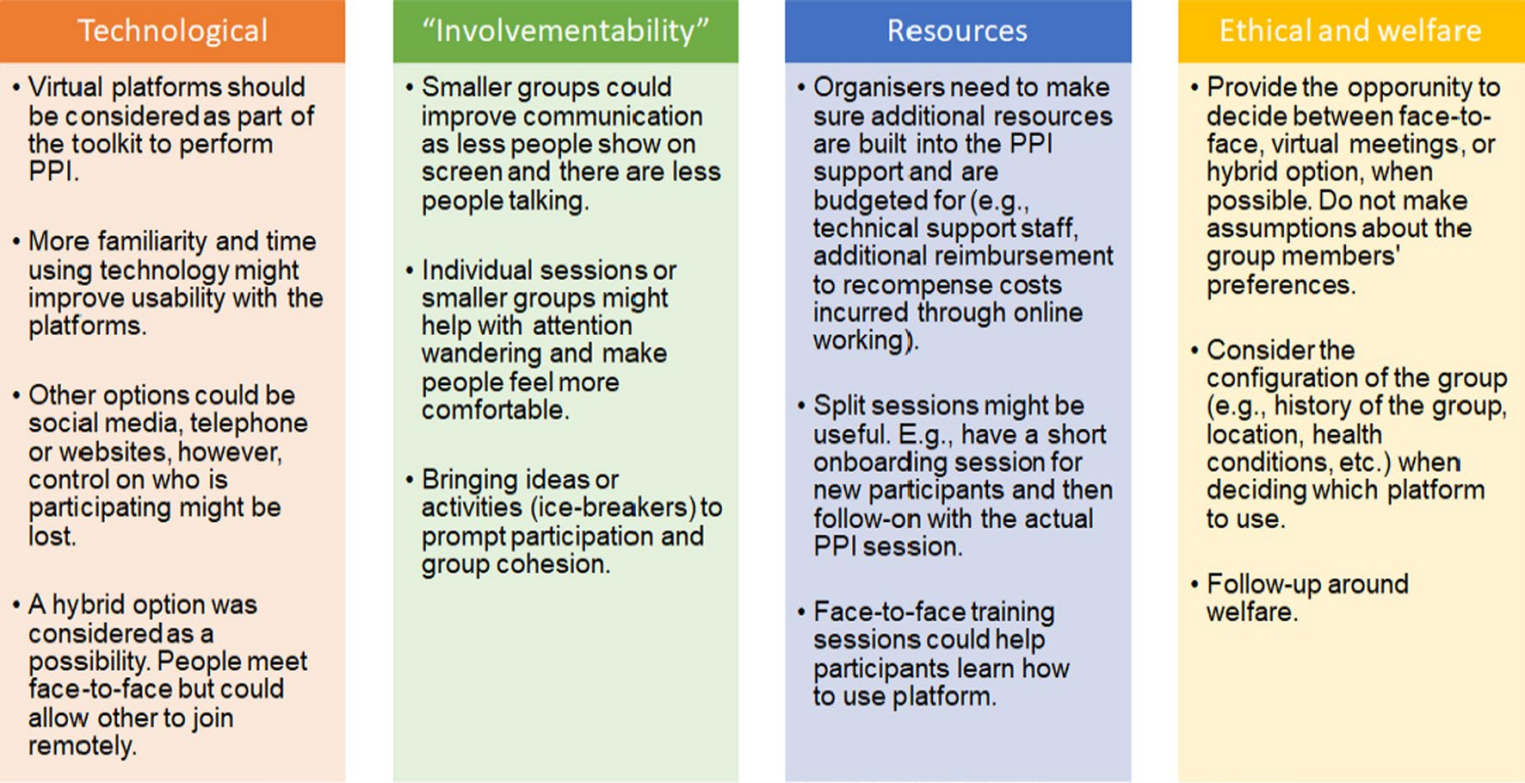
Tips to improve e-PPI meetings

By using the ‘E-nabling Digital Co-production’ Framework, several recommendations were obtained from the workshops’ discussions and mapped according to the areas of the framework. These recommendations were discussed with the MindTech Involvement Team during the data analysis and the final outcome is presented in Fig. 3.

## Discussion

### A new framework

The project aimed to consider e-PPI in a dementia-specific context and as a by-product led to refining existing guidance and co-producing the MindTech *‘E-nabling Digital Co-production’* Framework which is introduced here as a tool for researchers and PPI coordinators to help them identify and discuss challenges and opportunities provided by e-PPI.

Offering a step forward for thematic analysis, its four themes served well as the top-level codes, with insights from the participating groups mapped accordingly. We believe that the development process of the final framework (Fig. 1) is a good example of collaborative data analysis [21].

The framework development has adopted the term ‘co-production’, one described in terms of *Cobiquity’* by Williams, et al. [10], referring to the extent that it is frequently co-opted and potentially rendered meaningless. We have attempted to address issues of power and structural inequalities within the different categories and hope that the framework can be used by members of PPI groups and a journey, worthy of the term co-production, can be continued at a local level.

Although the four existing themes were used as the final codes, the framework is flexible enough to allow the inclusion of subcategories without modifying its content, meaning, and structure. For example, under the Ethical and Welfare code, three relevant subcodes were identified (Diversity and Inclusion, Digital Inclusion, Ethical Issues).

Although it was developed as a broad response to the transition to digital co-production (e-PPI) due to the pandemic this project shows that the framework is useful for specific populations and contexts, in this case for dementia research. We hope this means it will be readily used with other populations. Also, its adaptability to other situations makes it a suitable tool to study how e-PPI, and going forward, the complexities of blended meetings, will impact the involvement of the public in research, even more so in the COVID-19 context that is still evolving.

Likewise, the framework is not exclusive to e-PPI, but is rather a tool with the potential to consider how PPI is approached, both considering the current pandemic conditions, and going forward. It may help groups to explore their own preferences and the implications of different models of PPI within the post-pandemic transition. The experience of online meetings and increased familiarisation with digital platforms is likely to be built upon.

A previous systematic review reported 65 published frameworks for supporting, evaluating, and reporting PPI [23], however, none of them were targeting the digitalization of PPI. Although the authors of this review grouped those frameworks in five categories: (a) power-focused, (b) priority-setting, (c) study-focused, (d) report-focused, and (e) partnership-focused, we could not identify areas such as ‘technological*’* or ‘resources*’* that are included in our proposed framework. Furthermore, none of them included specific consideration of e-PPI or the move to e-PPI as part of a blended approach. For this reason, we consider that an extra category should be included in the proposed framework’s categorization associated to the approach on how to perform PPI (e.g., in-person or digital meetings) that could be termed ‘approach-focused’.

Regarding our findings in terms of the challenges and approaches of e-PPI, it is possible to identify both positive and negative opinions concerning digital co-production. However, ambiguities were also highlighted between the participants that opened deeper discussions, where a clear outcome from the assessment pros and cons is not obvious. For example, e-PPI was considered in one respect as a barrier in terms of the diversity and inclusion of the PPI groups if the same members are always attending the sessions, however, it was also mentioned that the virtuality could offer the opportunity to include others who have not engaged in PPI before. Within the dementia context, e-PPI offers carers the capacity to attend more meetings but simultaneously they may lose time away care responsibility.

Both workshop groups agreed that e-PPI acts as a barrier for communication and social interaction compared to experiences of meeting face-to-face but nonetheless offered an opportunity to re-evaluate the importance of meeting etiquette and communication styles which might provide a way into conversations for those who are less confident.

Therefore, e-PPI has a variety of pros and cons that must be evaluated as part of a context specific and co-produced response, to find the right solutions. Even with a retreat from the pandemic, e-PPI will remain embedded as a potential method to add value to existing approaches or to be considered as part of an evolving hybrid toolkit to perform PPI in research.

In addition to the differing opinions, two remarks were shared by the participating workshop groups. Firstly, that the removal of geographical constraints are useful to widen participation. Secondly that it saves resources. However, from an ethical perspective, these could still exacerbate existing inequalities. The transition to digital e-PPI has not occurred in a vacuum, with other external drivers and the recognition that diversity and inclusion within research is an area that requires multi-stakeholder action and commitment [1, 14]. Such topical debate dissects discussion around digital exclusion and wider inclusion in PPI and so practitioners need to explore the nuances of how e-PPI impacts this debate.

To increase social interaction, e-PPI methods could be adapted to include other approaches, potentially offering increased opportunity for this social interchange between public contributors and researchers and coordinators. This can be promoted by raising the awareness of the meeting chair to facilitate a more social atmosphere, taking an active role in focusing on inclusion of all meeting participants [17]. Also, other initiatives, such as online forums, could be more conducive to recreating the informal spaces of face-to-face meetings and overcome this vision of business-like sessions. This may further uncover ambiguities surrounding the purpose of PPI and the role of reciprocity in establishing relationships, where it is accepted that public contributors engage in health research with numerous and varying motivations [24]. As the literature on volunteering explores this phenomenon [25] it should be both recognised and reflected in efforts to reframe digital e-PPI that can mitigate the perception of a reduction to a transactional exchange, that has been highlighted.

New ethical challenges are ushered in within the digital domain, particularly the digital divide in populations, as the familiarity and access to the technologies remains inequitable, leading to an exclusion of vulnerable populations and some sectors of the society [14, 26]. Also, administrative considerations such as the consent form for recording or the reimbursement for public contributors, are topics warranting further discussion. This resonates with other considerations that whilst not arising during these workshops aspects such as internet security and data management are also important concerns.

The framework also offered recommendations to improve e-PPI, shown earlier in Fig. 3, and with some of these aligned with those suggested previously by Lampa, et al. [17].

Most of the insights overlapped between both workshops’ groups, suggesting that the results presented in this project are shared by the different populations involved. However, with the opportunity for both public contributors and organisers of e-PPI to consider these areas collaboratively or independently, it may serve to identify where there are different priorities and interpretations of costs and benefits associated with e-PPI. It is anticipated that this process itself could uncover both further areas to improve PPI and highlight power imbalances with regards how decisions on digital working are made.

### Dementia specific

We were interested to see if the framework could target specific insights for e-PPI in dementia research. Some of the remarks seem to be relevant for carers in general, regardless of the condition of the person being cared for, such as the pros and cons of convenience versus time away from caring as mentioned above. For example, on the positive side e-PPI seems to allow attending more sessions without being worried or stop attending due to their care role. However, by meeting online, virtual public contributors are missing a respite and supportive space that is present in face-to-face meetings, also they might be excluding those living alone or needing more support, and potentially more challenging to distinguish impacts that having a caregiver present may have on the level of participation of the person they are supporting.

For the dementia context, enabling those with cognitive impairment to take part is a more specific concern, hopefully leading to choices in the format of e-PPI to optimise involvement whether this is about being mindful abilities of participants to remember joining instructions, being mindful of levels of attention and concentration, or providing explicit cues to speaker. Degree of impairment will determine the level of support required, which may leads to the need for specialised training for facilitators, having additional supporters in the meeting, or other relevant potential solutions.

### Limitations and further projects

The project was conceived as a public involvement activity and for this reason, we did not explicitly collected demographic information, which is quite normal practice in PPI [27]. The workshops were biased to the views of those who were already engaged in remote communications, by necessity due to the pandemic. Results should therefore be used as an insight to improve future approaches to e-PPI in dementia research and other related contexts, rather than for their generalisability.

Due to the COVID-19 pandemic, difficulties were experienced with finding active PPI groups of PLwD, including those struggling to meet online. This led us to contact PPI groups known by the project team to be currently active. However, the Advisory group that participated in Workshop 2 was made of informal caregivers of PLwD, so a strong voice of PLwD for this project is missing. Furthermore, the time to perform the workshop with this group was shorter than the workshop with researchers and coordinators due to constraints of fitting within an existing meeting structure, which could have affected the volume and depth of insights identified from its members.

Only one digital platform was used (MS Teams), as it was the only one available to the institutions involved. Future studies could contribute by controlling the familiarity with online tools and by adding and comparing different digital platforms.

Future public involvement activities or research projects could test, use, and revise the framework to improve its usefulness. The flexibility that it affords, encourages individual groups to explore their own journey and co-produce a bespoke response, with the potential to adapt to an increasing body of involvement methods, including blended approaches that include a digital element. As such it could be beneficial for researchers, organisations and individuals undertaking future PPI activities.

Regarding dementia digital PPI, it would be beneficial to undertake further research on a larger scale and possibly incorporate comparisons between types of dementia to explore if this has an impact on preferences. This framework could be used on a micro level such as a PPI group with particular social and health needs to better understand the digital preferences of the group and consequently have better outcomes for sessions. On the other hand, the framework could be utilised on a macro level to undertake national studies to understand the PPI digital working needs of those with different health conditions. As the stages of the pandemic change and restrictions are lifted and face-to-face working is fully or partly resumed, it is imperative that those facilitating PPI activity are aware of the impact that e-PPI working has had upon the preferences among public contributors and the potential impact on power dynamics. Finally, with potentially increased ability to facilitate face-to-face PPI activity, future research regarding e-PPI could include those not currently involved in digital working for a broader understanding. It would be recommended future research continues to explore e-PPI (and blended approaches) in both dementia and other conditions to gain a clearer understanding of how we can better facilitate future e-PPI working in these ever-changing times.

## Conclusion

The ‘E-nabling Digital Co-production’ Framework that was developed through this public involvement activity was useful in advancing understanding of the issues and opportunities regarding e-PPI. It also helped identify specific insights for facilitating PPI in dementia research. The framework and approach to e-PPI described here could be generalised to further projects. This project also provides an example of a journey of co-production in developing PPI practice.

## Supplementary Information

**Additional file1**: **Table S1.** Overview of digital considerations. It shows the MindTech Involvement Team’s first overview of the primary areas that were impacted by the shift to e-PPI and the local actions taken to mitigate challenges and harness the potential from working digitally.

**Additional file2**: **Project Information Sheet.** It describes the project and includes the contact information of the Project Leads given to the participants before their involvement in the project.

**Additional file3**: **Semi structured Questions Guide.** It shows the questions that were used to guide the workshops, this document was given beforehand to the participants.

## Data Availability

Data sharing is not applicable to this article as no datasets were generated or analyzed during the current study.

## Abbreviations

COVID-19: Coronavirus disease 2019
PPI: Patient and public involvement
e-PPI: Electronic/digital patient and public involvement
ICT: Information and communication technologies
DISTINCT: Dementia: intersectorial strategy for training and innovation for current technology
PLwD: People living with dementia
MS Teams: Microsoft teams
DHIs: Digital health interventions
WFH: Working From Home
NIHR: National Institute for Health and Care Research
