# Supplementary material for: Insights on conducting digital patient and public involvement in dementia research during the COVID-19 pandemic: supporting the development of an “E-nabling digital co-production” framework
Source: Res Involv Engagem. 2022 Jul 26;8:33. doi: 10.1186/s40900-022-00371-9 (PMC9321274; doi:10.1186/s40900-022-00371-9)
Supplement: Supplementary file 1 — Additional file1: Table S1. Overview of digital considerations. It shows the MindTech Involvement Team’s first overview of the primary areas that were impacted by the shift to e-PPI and the local actions taken to mitigate challenges and harness the potential from working digitally. [file 40900_2022_371_MOESM1_ESM.pdf]

Additional file 1: Table S1. Overview of digital considerations.

**TECHNOLOGICAL CONSIDERATIONS**	**Preference**<br>• Consider other less technological options, based on individual preferences, including: One to one phone calls may work best for those with limited access to the internet, or for more in-depth discussions.<br>• Teleconferences could also be an option, if a group discussion would be helpful but internet access is a challenge.<br>**Access**<br>• Does the channel, e.g. MS Teams, Zoom or MIRO etc. work where people are using different devices, e.g. smart phones, need for 2 screens etc.?<br>• Internet connection<br>**Aptitude**<br>• How familiar are participants with remote communication and what are people's preferences?<br>• Need to balance limitations on inclusivity (e.g. using people with more technological experience) against ensuring that sessions can operate smoothly and people able to contribute effectively.
**ONLINE FACILATION CONSIDERATIONS**	**Developing roles**<br>• Clear roles needed from the outset<br>• Power-dynamics and how recruiting a PPI Co-facilitator can provide supportive environment<br>• Consider 2nd Facilitator, specifically looking at inclusion through monitoring online chat and other channels used to communicate<br>**Online Ground Rules and Etiquette**<br>• Establish ground rules from the offset, with clear mechanisms to enable participation from all involved<br>• Recognise and respect that standard behavioural standards/expectations of digital meetings will differ from face-to-face, e.g. acknowledge potential interruptions due to caring responsibilities or technology, allowing people the space to respond and re-join the group<br>**Time management**<br>• Allowing enough time to cover agendas and topics<br>• Importance of communicating and sharing information beforehand to maximise time gaining insight from PPI during the sessions<br>• Recognising PPI participants may not be able or wish to stay longer than meeting end time.<br>• Recognising PPI participants may have responsibilities and may not be able to provide involvement at short notice
**CO-PRODUCTION CONSIDERATIONS**	**Workshop numbers**<br>•Depending on the configuration and systems used, including the ability to facilitate breakout sessions, participant numbers should reflect the additional challenges and requirements of digital PPI.<br>**Co-Design preparation**

	• Preparation for a digital workshop requires additional skills and time dedicated to creating a user-friendly experience.<br>• Issues with trying and replicate ½ day face-to-face workshop on digital<br>• Breaking down activities into smaller segments and ensure a clear plan for the session guided by facilitator<br>• Consider sending out documents to review beforehand if appropriate, instead of trying to present them all in the platform.<br>**Collaborative working**<br>• Different activities could include Synchronous (where everyone is in contact at a specific time, mimicking a typical workshop) or asynchronous input, e.g. MIRO allows you to post outside the session forum/MS Teams for specific project or hashtag where people can post at different times more suitable for them. Also providing a video tutorial of how to use the platform to help people familiarise
**RESOURCE CONSIDERATIONS**	**Additional resources**<br>• As well as the cost of coproduction platforms themselves, these could include phone credit/printing etc./software/budgeting for an increased frequency but shorter meetings<br>• Cost and time of sending equipment and resources to people who need this to partake<br>**Workshop numbers**<br>• Depending on the configuration and systems used, including the ability to facilitate breakout sessions, participant numbers should reflect the additional challenges and requirements of digital PPI.<br>**Training and Opportunities**<br>• Where a PPI Member has a role within the Research Team, time and resources should be afforded to their training and development in digital coproduction.
**ETHICAL & WELFARE CONSIDERATIONS**	**Impacts of digital PPI on welfare**<br>• Change from face to face PPI impacting of wellbeing – isolated, anxiety, depression, lack of movement; On the other hand some may feel digital PPI is a better fit for them. Creating friendly space and social aspects.<br>• Certificates and follow ups of project progress to promote wellbeing and meaning<br>• Consider the need for additional breaks to recognise impact of working online – (consider those new to Techognoly use or health issues of the public & patient that may require adequate breaks)<br>**Consent & Safeguarding**<br>• Consider use of Peer Facilitator in next virtual room (or on different platform- being clear that run by separate/independent organisation<br>• Considering how support mechanisms have been adapted for a digital environment, e.g. use of Peer Support in an alternative virtual room, or accessible by pone before, during and after a PPI session<br>**Data security**<br>• Asking if we are ok to have session recorded<br>• Addressing concerns about privacy on platforms and where research will be shared
