# Supplementary material for: Insights on conducting digital patient and public involvement in dementia research during the COVID-19 pandemic: supporting the development of an “E-nabling digital co-production” framework
Source: Res Involv Engagem. 2022 Jul 26;8:33. doi: 10.1186/s40900-022-00371-9 (PMC9321274; doi:10.1186/s40900-022-00371-9)
Supplement: Supplementary file 2 — Additional file2: Project Information Sheet. It describes the project and includes the contact information of the Project Leads given to the participants before their involvement in the project. [file 40900_2022_371_MOESM2_ESM.pdf]

Additional file 2. Project Information Sheet

PROJECT INFORMATION AND INVITATION TO CONTRIBUTE

1. Title

How to approach Public and Patient Involvement (PPI) in dementia research through technologies? Feedback from *experts by experience.*

2. Invitation paragraph

Are you interested in contributing to enhance knowledge and determine better practices on the use of Information and Communication Technologies (ICTs) as an approach to involve public as part of dementia research teams? We are planning online workshop sessions to discuss about our experiences on the use of communication technologies as a facilitator tool to participate in dementia research as *experts by experience*.

No previous experience is needed regarding participation in dementia research, but at least you must have had minimum experience on using ICTs to communicate with others. However, you do not need to be an expert on ICTs, neither concerning their use to participate in research.

3. What is the purpose of the study?

Due to the COVID-19 pandemic, the involvement of patients and public in research has been challenged because of social distancing, lockdowns and reduced physical contact. Now, after more than one year since the beginning of this situation, the implementation of ICTs

has been regarded the most used way to tackle these challenges, however, due to the promptness of the reaction, we might not have had the opportunity to ask the public involved how they feel using these new approaches, if the ICTs are user-friendly, or if ICTs are successful tools to get involve in shaping or guiding dementia research.

Sharing our experiences and opinions, will not only enhance how we use ICTs in dementia research during COVID-19 pandemic, but also for facing other constraints that might diminish the public engagement in regular circumstances, such as access to rural and remote areas, limited availability of services and health professionals, distance from healthcare services, poverty, PPIs Representatives' health conditions, such as fatigue, mobility restrictions, work or family commitments, limited budget for research with PPI, etc.

For this purpose, we will like you to get involve as an *expert by experience* to contribute on this discussion and propose ideas and tips on how to improve the involvement of the public on dementia research.

4. **Why have I been invited?**

We consider you as an *expert by experience* that can share your valuable opinion and knowledge about the topic being discussed. Even if you have not been part of a dementia research team before, you can provide suggestions around how ICTs can be adapted, or how we can adapt them, to have a better and more friendly experience supporting dementia research to keep public involve.

5. **Do I have to take part?**

It is up to you to decide whether or not to take part. You are free to withdraw at any time without giving a reason.

6. **What will happen to me if I take part?**

If you decide to take part of the workshops, you will be invited to a virtual meeting. Beforehand, you would receive information about the project, instructions to use the platform that will be used to run the workshops, general questions that will be used to start the discussion, and an opportunity to ask questions to the organizers. During the online workshops you will be sharing with other people and have the opportunity to discuss about the specific topics planned for that day. One of the organizers will be leading the discussions and will guarantee that all the participants have their chance to speak and give feedback. Meetings will not be recorded, and it is not necessary to activate the camera/video of the platform. You will also have the opportunity to share your ideas through the chat box, if you find it necessary. Feedback with the notes taken from the organizers will be sent to all the individuals involve. The aim is that together we could elaborate some tips and ideas to enhance the use of ICTs to be engage in dementia research planning and development.

7. **What are the possible disadvantages and risks of taking part?**

You might get exhausted during the workshops, but no other risks or disadvantages are expected. However, you are free to leave the session at any time. Also, you can take a break during the session and re-join if you consider so.

9. **What are the possible benefits of taking part?**

You will receive no direct benefit from taking part in this project. However, you may have the option to be included as part of the contributors in any further material or publication that could be elaborate with the information discuss during the workshops. The notes taken during the project, will enhance the knowledge on ICTs to improve public involvement in dementia research.

10. Will my taking part in this project be kept confidential?

Yes, your participation will be kept confidential. This is not a study, is a Public Involvement contribution. Any information gather during the project, could be presented in general terms in further publications or materials, but it will never be connected to any of the contributors participating at the workshops. We will not be asking for personal details or sociodemographic information.

11. What will happen to the information gather from the workshops?

One of the organizers will be taking notes, this information could be published as a report, guideline, or any other publication. Your participation will be recognized in any further material resulting from the project. You will not be identified in any of the reports or publications following the project, unless you explicitly suggest for it.

12. Who is organising and funding the project?

This project is part of DISTINCT: "Dementia: Intersectorial Strategy for Training and Innovation Network for Current Technology" which supports fifteen Early Stage Researchers (ESRs) across Europe, who are carrying out research projects aiming to improve the lives of people with dementia and their carers through technology. DISTINCT

is funded by the Marie Skłodowska Curie Actions Innovative Training Network H2020-MSCA-ITN, under grant agreement number 813196

The project is also been organized by MindTech which is a national centre focussing on the development, adoption and evaluation of new technologies for mental healthcare and dementia and funded by the National Institute for Healthcare Research.

13. **Who has reviewed the project?**

Research Ethics Approval to conduct this project is not required as it is a public engagement activity. **Remember, this is not study, is a voluntary contribution to engage in a Public and Patient Involvement activity.** Faculty of Medicine and Health Science Research Ethics Committee reference no. 255-0521.

Contact for Further Information

MSc. Mauricio Molinari Ulate

Mobile: +34653327757

E-mail: mmolinari@ides.es

Rebecca Woodcock

Patient and Public Involvement Co-ordinator: Mental Health & Technology Research

E-mail: rebecca.woodcock@nottingham.ac.uk

Note: You will be given a copy of this information sheet by email. Also, we offer the possibility to send a copy by post upon previous request.
