# Supplementary material for: Insights on conducting digital patient and public involvement in dementia research during the COVID-19 pandemic: supporting the development of an “E-nabling digital co-production” framework
Source: Res Involv Engagem. 2022 Jul 26;8:33. doi: 10.1186/s40900-022-00371-9 (PMC9321274; doi:10.1186/s40900-022-00371-9)
Supplement: Supplementary file 3 — Additional file3: Semi structured Questions Guide. It shows the questions that were used to guide the workshops, this document was given beforehand to the participants. [file 40900_2022_371_MOESM3_ESM.pdf]

Additional file 3. Semistructured Questions Guide

- What strengths do you identified regarding the use of online digital platforms for PPI in dementia research (e.g., platforms like Zoom, Teams, GoToMeeting)?
- Do you have the necessary equipment for the meetings?
- Do you struggle using the equipment or the online platforms?
- Do you feel these platforms are user-friendly?
- Do you prefer online or face-to-face meetings regarding PPI in dementia research?
- How confident do you feel using these technologies for PPI in dementia research?
- Do you feel included or involved in the discussion or is it less engaging or inclusive?
- How safe do you feel using the technologies for engaging in dementia research?
- How supported do you feel using online approach? Do you feel supported by the people hosting the meetings?
- Do you require any additional support by others (e.g., caregivers)?
- How emotional support could be managed during online PPI in dementia research? Or how do you feel about that and how do you think this must be managed?
- Have you been able to do more PPI, or has it been the same?
